# Mounting *Drosophila* pupae for laser ablation and live imaging of the dorsal thorax

**DOI:** 10.1016/j.xpro.2022.101396

**Published:** 2022-05-14

**Authors:** James T. O’Connor, Erica K. Shannon, M. Shane Hutson, Andrea Page-McCaw

**Affiliations:** 1 Department of Cell and Developmental Biology, Vanderbilt University, Nashville, TN, USA; 2 Program in Developmental Biology, Vanderbilt University, Nashville, TN, USA; 3 Department of Physics and Astronomy, Vanderbilt University, Nashville, TN, USA; 4 Department of Biological Sciences, Vanderbilt University, Nashville, TN, USA; 5 Institute for Integrative Biosystems Research and Education, Vanderbilt University, Nashville, TN, USA; 6 Vanderbilt-Ingram Cancer Center, Vanderbilt University, Nashville, TN, USA

**Keywords:** Cell Biology, Developmental biology, Microscopy, Model Organisms

## Abstract

This protocol describes the preparation of *Drosophilamelanogaster* pupae for laser ablation and live imaging of the notum (dorsal thorax). Because the pupa is stationary, it can be continuously live imaged for multiple days if desired, making it ideal for studying wound signaling and repair, from before laser ablation through wound closure. In this protocol, we demonstrate the processes of staging, partially dissecting, mounting, wounding, and live imaging the pupal notum, with the wounding occurring during the live imaging process.

For complete details on the use and execution of this protocol, please refer to O’Connor et al. (2021b).

## Highlights

• The *Drosophila* pupa is an attractive model to study wound repair *in vivo*
• The dorsal thorax epithelium is accessible after partial dissection of the pupal case
• Pupae are mounted on a cover glass and wounded by laser ablation
• Pupae are imaged live during wounding through wound closure to analyze signaling/repair

## Abstract (editor highlights)

Publisher's note: Undertaking any experimental protocol requires adherence to local institutional guidelines for laboratory safety and ethics.

## Graphical abstract

**Figure:**
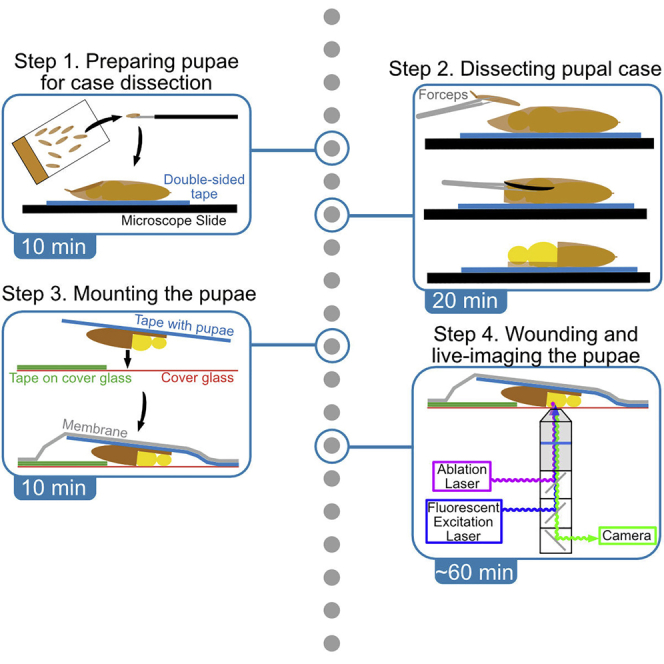

## Teaser

This protocol describes the preparation of *Drosophilamelanogaster* pupae for laser ablation and live imaging of the notum (dorsal thorax). Because the pupa is stationary, it can be continuously live imaged for multiple days if desired, making it ideal for studying wound signaling and repair, from before laser ablation through wound closure. In this protocol, we demonstrate the processes of staging, partially dissecting, mounting, wounding, and live imaging the pupal notum, with wounding occurring during the live imaging process.

## Before you begin

The protocol below uses pupae with the following genotype, but could use *Drosophila* of any genotype:

*ActinP-GCaMP6m / +; pnr-Gal4, UAS-mCherry.NLS,TubP-Gal80*^*TS*^*/ +*.

This genotype will yield pupae that ubiquitously express the calcium indicator GCaMP6m (Chen et al., 2013), and express the red nuclear marker mCherry.NLS (Caussinus et al., 2008) in the *pnr* domain (Calleja et al., 1996) under temperature-sensitive control using Gal80^TS^ (McGuire et al., 2003). In the study by (O’Connor et al., 2021b), *UAS-RNAi* lines were crossed into this system to knock down genes of interest exclusively in the *pnr* domain while maintaining an adjacent internal control. To inactivate Gal80^TS^ and activate *UAS*-mediated gene expression, the experimental samples were kept at 29°C for 4–5 days before experimentation. Note that Preparation one is at 29°C, and at that temperature, pupae develop approximately 1.3× faster than if kept at 25°C (Bainbridge and Bownes, 1981; David, 1988).

### Setting up a laser ablation system for wounding

**Figure 1:**
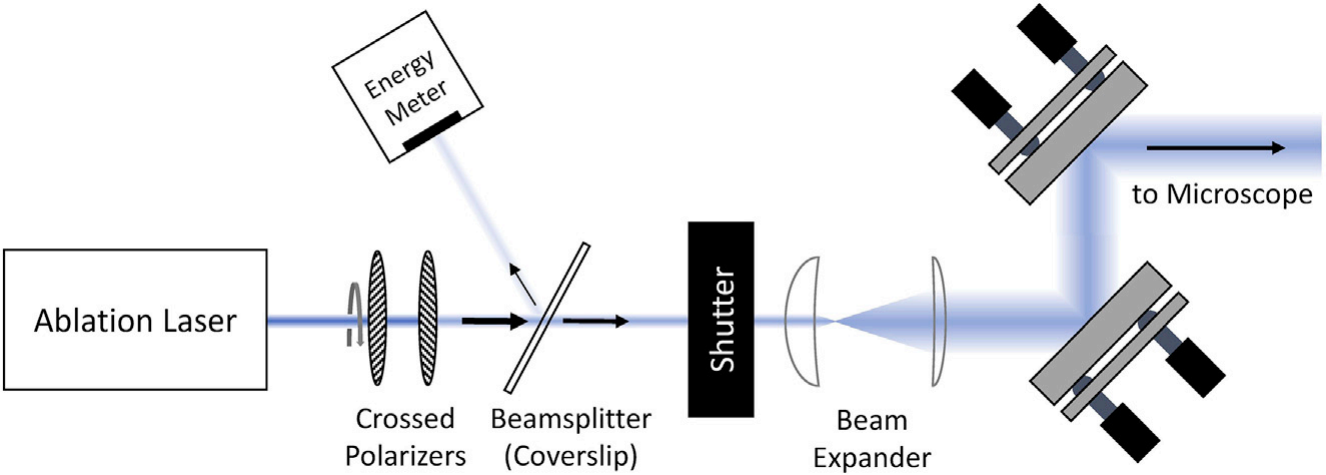
Schematic of the optical path from ablation laser to an entry port on the microscope Left to right, the pair of crossed polarizers provide control of delivered laser intensity: rotation of the primary axis of the first polarizer reduces transmission of light through the fixed primary axis of the second polarizer according to Malus’ Law. Next, a coverslip serves as a beam-splitter to reflect a small portion of the laser light to an energy meter, which allows continuous monitoring of laser pulse energy. Then, a mechanical shutter controls when laser light is delivered to the microscope, and a pair of converging lenses with different focal lengths are used to expand and recollimate the laser beam. All optics to this point are mounted on a single rail to facilitate alignment. Finally, the beam is reflected from two or more flat mirrors on kinematic mounts to enable steering the beam for proper alignment into the microscope. On most microscopes, there are multiple ports available for introducing an external beam, so details of the beam path from entry port to objective can vary. For example, a Zeiss Axiovert 135 / LSM-410 microscope uses the bottom port and requires an extra converging lens to form a pair with the tube lens inside the microscope between this port and the objective. In contrast, a newer Nikon Eclipse Ti2 microscope introduces the expanded and nearly collimated beam of the ablation laser into a back epi-illumination port. Optics interior to the microscope typically include a dichroic mirror, which must be chosen appropriately so that ultraviolet light from the ablation laser reaches the back aperture of the imaging objective. The objective should also be chosen for its ability to transmit ultraviolet light, for example a 40×/1.30 Plan-Neofluor (ZEISS) or a 40×/1.30 NA S Fluor (Nikon) objective.

The setup for laser wounding requires coupling an ablation laser into a confocal microscope. The equipment and set up has been described previously (Kiehart et al., 2006). For the protocol shown here, the ablation laser is the 3rd harmonic of a Q-switched Nd:YAG laser (wavelength 355 nm; pulsewidth 3 ns), but other pulsed ultraviolet lasers could be substituted. The key components of the optical path from ablation laser to microscope are summarized in Figure 1.

To properly align the ablation laser into the microscope, first adjust the flat steering mirrors so that the beam enters the microscope through the middle of the chosen port and exits through the objective turret set to a blank position. Since the beam is in the near-ultraviolet, it is not visible to the human eye; however, a white business card or piece of paper inserted into the beam will fluoresce at blue wavelengths and allow beam visualization. Once the beam is roughly centered on the blank turret position, close the shutter, and rotate the turret to the desired objective. Then image an appropriate target, such as a slide of fluorescein-stained agarose gel as described in (Kiehart et al., 2006), and briefly open and close the shutter to ablate a spot on the target. Use the microscope’s stage to move to fresh regions of the target and adjust the crossed polarizers until such ablations leave a ∼1-μm-diameter mark. Then adjust the steering mirrors until the ablation mark appears consistently in the middle of the field of view.

The ablating beam is now properly aligned in the xy-plane, but most objectives have chromatic aberration at ultraviolet wavelengths that requires adjustment of the ablation laser’s convergence/divergence to focus the ablation laser at the imaging plane. To make this adjustment, put the ablation laser in single-shot mode, open the shutter, and deliver single laser pulses to fresh regions of the target while reducing laser energy until the ablation mark is just barely visible. Then make slight adjustments to the separation between the two lenses of the beam expander and ablate fresh spots. The best z-alignment is the separation that yields the strongest ablation marks. Alignment of the ablation laser in x, y and z should be rechecked periodically.

### Staging the pupae (method one)

**Timing: 1–6 h before**

Rationale: Staging the pupae is necessary to get reproducible results. In this protocol, we aim to experiment exclusively on pupae between ∼12–18 h after puparium formation (APF) at 29°C. A little before 12 h APF, the pupa undergoes apolysis – the process where the epidermis pulls away from the pupal case – and the body plan takes shape, and the head everts (Bainbridge and Bownes, 1981). After these events, the pupa can be removed from its case. By 18 h APF, the notum will no longer lie as flat against the cover slip, and the cells that form the thoracic bristles begin to be visibly distinct from their neighboring cells, potentially interfering with imaging of the notum. Staging is important because pupae look similar by eye between 12 and 48 h APF and therefore are difficult to identify.

Need: Dissection scope, Sharpie permanent marker, vial of mixed 3^rd^ instar larvae and pupae.

**Figure 2:**
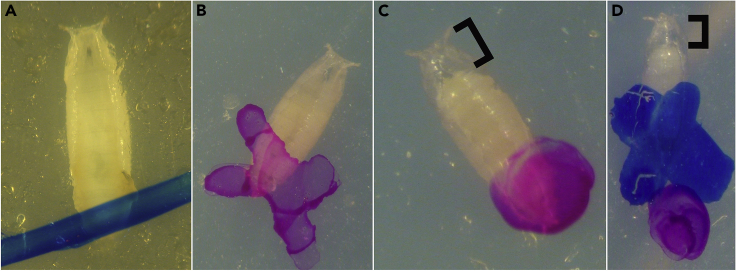
Identifiable stages of pupal development (A) White prepupae, ∼0–1 h APF at 29°C, which are white like 3rd instar larvae but are stationary and display everted anterior spiracles. (B) Brown prepupae, ∼1–12 h APF at 29°C, where the pupal case has tanned, but the larval body remains adhered to the inside of the pupal case. (C) Head-everted pupae, ∼12+ hrs APF at 29°C, where the head of the pupa has everted and the adult body plan has formed. (D) A head-everted pupa, which was previously demarcated as a brown prepupa less than 6 h prior (blue X), is therefore less than 18 h APF and suitable for experimentation. (C and D) Brackets denote an easily identifiable gap that appears between the head of the pupa and the anterior spiracles.
  b. Brown prepupae, ∼1–12 h APF at 29°C, P2–P4. The pupal case has tanned, but the larval body remains adhered to the inside of the pupal case (see Figure 2B). Because apolysis defines the transition from prepupa to pupa, these animals are technically still prepupae. Mark the outside of the vial for these brown prepupae with a second identifying mark, such as an “X” (see Figure 2B).
  c. Head-everted pupae, ∼12+ hrs APF at 29°C, P4(ii) onward, where the head of the pupa has everted and the adult body plan has formed. A small gap has appeared between the head of the pupa and the anterior spiracles (brackets in Figures 2C and 2D). Mark the outside of the vial for these pupae with a third identifying mark, such as a “•” (see Figure 2C).
2. Return the vial to its original temperature (29°C, here). Make note of when these pupae were marked.
3. No more than 6 h later, when you are ready to dissect the pupae, re-evaluate the pupae that had previously fallen into the category of brown prepupae marked with an “X”. If they are now head-everted pupae (as in Figure 2D), they can be used for experimentation.
  a. Any head-everted pupa that was a brown prepupa 6 h prior now falls between 12–18 h APF, and therefore is at the proper time point.

***Note:*** If your experiment requires a more precise time point, say 12–15 h APF, then the brown prepupae would need to be re-evaluated no more than 3 h after initial marking. This staging process is amenable to your experiment’s needs.

1. Under a dissection scope, identify and classify each pupa in a vial according to three categories:
  a. White prepupae, ∼0–1 h APF at 29°C, P1–P2 (Bainbridge and Bownes, 1981), which are white like 3^rd^ instar larvae but are stationary and display everted anterior spiracles. Mark the outside of the vial where the white prepupa is adhered using a permanent marker to make a small identifying mark, such as a “/” (see Figure 2A).

4. To stage animals that are still prepupae, as well as the new prepupae that were previously larvae, repeat steps 1–3, using a different colored permanent marker.

### Staging the pupae (method two)

**Timing: 12–17 h before**

Need: Dissection scope, permanent marker, vial of mixed 3^rd^ instar larvae and pupae.

5. Under a dissection scope, identify, classify, and mark **every single** pupariated animal (prepupae and pupae) in the vial according to the same categories as Preparation one, Method one. This time it is vital to be sure all pupae in the vial are marked and none have been accidentally missed. If a pupa is unmarked at this stage, then later in step 7, one may mistake a much older pupae for a newly formed one, and accidentally use a pupa that is far older than 12–18 h APF.
6. Return the vial to its original temperature (29°C, here). Make note of when the animals were marked.
7. No more than 17 h later, when you are ready to dissect the pupae, re-evaluate all the pupae in the vial. If they were previously white prepupae and are now head-everted pupae, or if they were previously unmarked (because they were larvae during previous staging) and are now head-everted pupae, they can be used for experimentation.
  a. Any head-everted pupa that was a white prepupa 12–17 h prior now falls between 13–18 h APF, and therefore is at the proper time point.
  b. Any head-everted pupa that was previously unmarked must have been a larva at the time of marking, now falls between 12–17 h APF, and therefore is at the proper time point.

### Staging the pupae (method three)

**Timing: 12–17 h before**

Need: Dissection scope, permanent marker, several vials or bottles of mixed 3^rd^ instar larvae and pupae.

Note that although this method is simpler, it requires about 6 times as many animals as the previous two methods.

8. Under a dissection scope, mark all the white prepupae observed on the sides of the vials and/or bottles.
9. Return the vials/bottles to their original temperature (29°C here). Make note of when these white prepupae were marked.
10. Between 12–17 h later, all the marked animals will be 12–18 h APF.

## Key resources table

**Table:** 

REAGENT or RESOURCE	SOURCE	IDENTIFIER
**Experimental models: Organisms/strains**		

*D.melanogaster* (pupae, not differentiated by sex): *ActinP-GCaMP6m / +; pnr-Gal4, UAS-mCherry.NLS,* T*ubP-Gal80*^*TS*^*/ +*	O'Connor et al., 2021b	N/A
*D.melanogaster* (pupae, not differentiated by sex): *ActinP-GCaMP6m*	O'Connor et al., 2021b	
*D.melanogaster* (pupae, not differentiated by sex): *pnr-Gal4*	Calleja, et al., 1996	FlyBase ID: FBst0025758
*D.melanogaster* (pupae, not differentiated by sex): *UAS-mCherry.NLS*	Caussinus, et al., 2008	FlyBase ID: FBst0038424
*D.melanogaster* (pupae, not differentiated by sex): *TubP-Gal80*^*TS*^	McGuire, et al., 2003	FlyBase ID: FBst0007017

**Software and algorithms**		

FIJI	Schindelin, et al., 2012	https://imagej.net/Fiji
Affinity Designer	Affinity Serif	https://affinity.serif.com/en-us/designer/

**Other**		

Zeiss Stemi 2000 Stereo Dissecting microscope	ZEISS	SKU: SP-STEMI2000-TS2
Sharpie permanent markers in multiple colors	Sharpie	Item # 30072
Double Frosted Microscope Slide (25 × 75 × 1.0 mm)	Fisher Scientific	Cat: 22-034-486
Cover Glass (35 × 50 No. 1)	Fisher Scientific	Cat: 125485R
Fisherbrand™ Fine Precision Probe	Fisher Scientific	Cat: 12-000-153
#5 or #55 Forceps	Dumont	#5 or #55
Double-Sided Tape	Scotch	Cat: 665
YSI Standard Membrane Kit	YSI	MFR# 5775
Wide *Drosophila* Vials, Polypropylene	Genesee	Cat: 32-114
Zeiss LSM410 Scanning Confocal Microscope	ZEISS	LSM410
Continuum Minilite II Q-switched Nd:Yag Laser	Continuum	Minilite II
Optical Rails and Rail Carriers	Newport	
Polarizing Cube Beamsplitters / Polarizing Prism	Thorlabs	GLB5-405
Polarizer Mounts	Thorlabs	PRM05GL5
Photodiode Energy Sensor	Ophir Optical	PD-10
Nova Handheld Energy Meter	Ophir Optical	Nova 7Z01500
Shutter	Uniblitz	VS25
Shutter Drive Controller	Uniblitz	VMM-D1
Converging Lens #1	Thorlabs	Part #LA1085
Lens Mount #1	Thorlabs	LMR18
Converging Lens #2	Thorlabs	Part #LA1401
Lens Mount #2	Thorlabs	LMR2
Flat Mirrors	Newport	20D20AL.2
Agarose	Sigma-Aldrich	A9539
2′,7′-dichlorofluorescein	Sigma-Aldrich	D6883
Type DF Immersion Oil	Cargille Laboratories	Cat# 16242

## Step-by-step method details

### Removing pupae from the vial and preparing pupae for case dissection

**Timing: ∼10 min**

This section will get properly staged pupae out of their vial, onto double sided tape, and ready for dissection.

**Figure 3:**
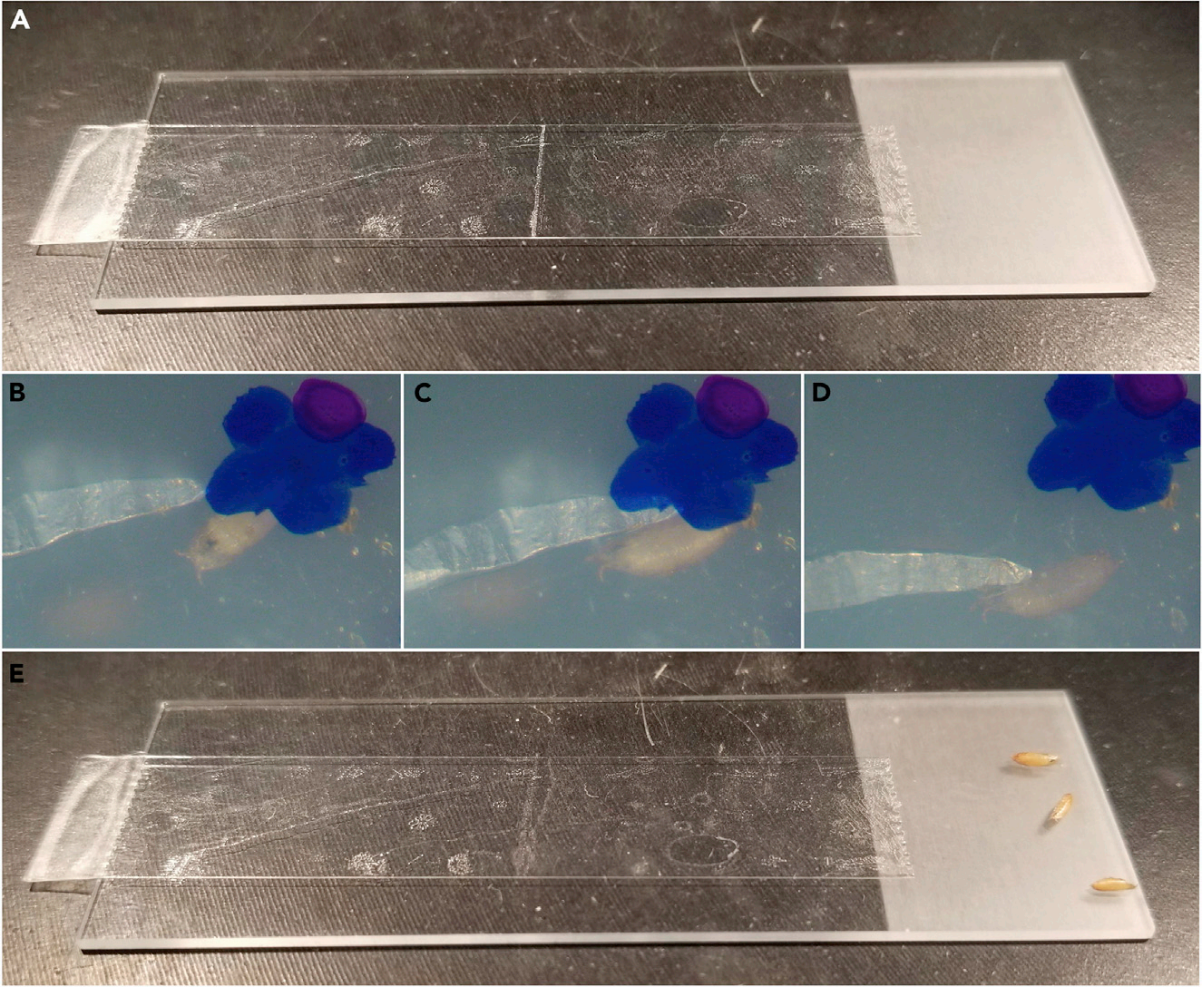
Removal of pupae from a vial (A) A piece of double-sided tape attached lengthwise on a microscope slide, with an overhang to easily remove later, but not completely covering the slide so there is sufficient space remaining to place pupae after they have been removed from the vial. (B and C) A dull flat-ended probe can gently pry a pupa away from its vial. (D and E) (D) When the pupa is pried from the vial, it will generally remain lightly adhered to the probe, allowing it to easily be moved to the empty region of the microscope slide (E).

**Figure 4:**
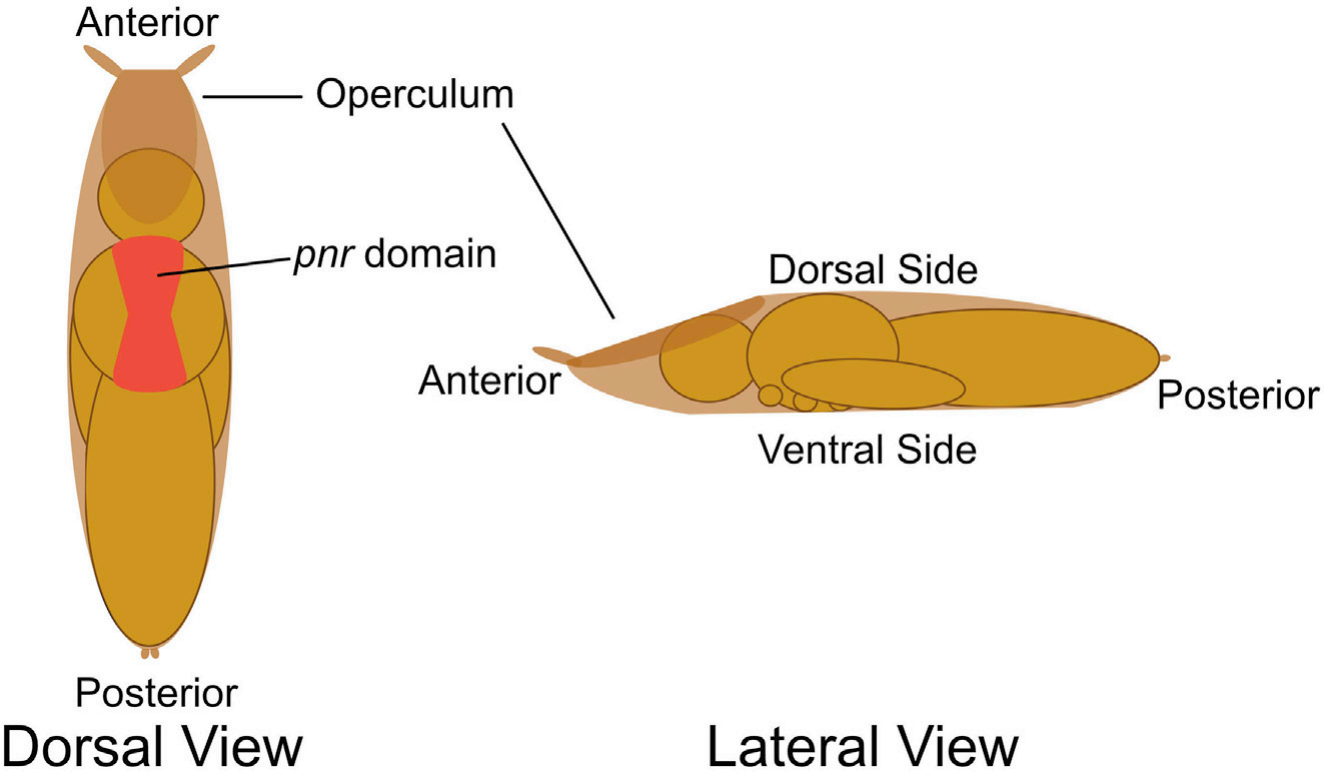
Anatomical diagram of a pupa Dorsal and lateral views of a *Drosophila* pupa show the adult head, thorax, and abdominal body sections contained within the pupal case. The operculum (the flattened exit from which the adult emerges, indicated here as the darkest brown region) and the attached anterior spiracles (wide “horns” protruding from the operculum) can be used to easily identify the anterior of the pupa from the posterior. The *pnr* expression domain of the dorsal thorax (notum) is denoted in red.

1. Place a piece of double-sided tape lengthwise on the center of a microscope slide as shown in Figure 3A, so that the tape begins about one-half inch onto the slide and ends with a small tab overhanging the slide’s edge. The tab will allow the tape to be easily lifted from the slide later.

2. Use the dull flat-ended probe to very gently pry several staged pupae off the side of the vial. Note that the ventral side of the pupae is adhered to the vial, with the anterior side facing down and left. See Figure 4 to orient dorsal, ventral, anterior, and posterior sides.
  a. Very gently maneuver the flattest part of the probe underneath the pupal case, then lift it away from the vial, as shown in Figures 3B–3D.
    **CRITICAL:** The posterior end of the pupa is generally more resilient to being prodded than the anterior side, but the entire pupa is fragile. If the pupa is popped, it should be discarded.
  b. When the pupa lifts away from the vial, it should stick to the probe. Carry it to and the microscope slide and collect several pupae on in the region not covered with double-sided tape, as shown in Figure 3E.

3. Use the fine-tipped forceps to gently grasp the pupa between the anterior spiracles, where the gap between the pupa’s head and its case has formed (Figure 5A). The anterior spiracles can be identified as the wide “horns” extending out from the operculum (see Figure 4); the black larval mouth parts, visible in this gap between the pupa’s head and its case, can also be used to identify the anterior side and demarcate where to pinch with forceps. Then place the pupa ventral side down onto the double-sided tape near the center of the tape, leaving equal space anterior and posterior to the pupae, and enough space laterally to eventually grab and remove the tape from the microscope slide later (about one inch).

**Figure 5:**
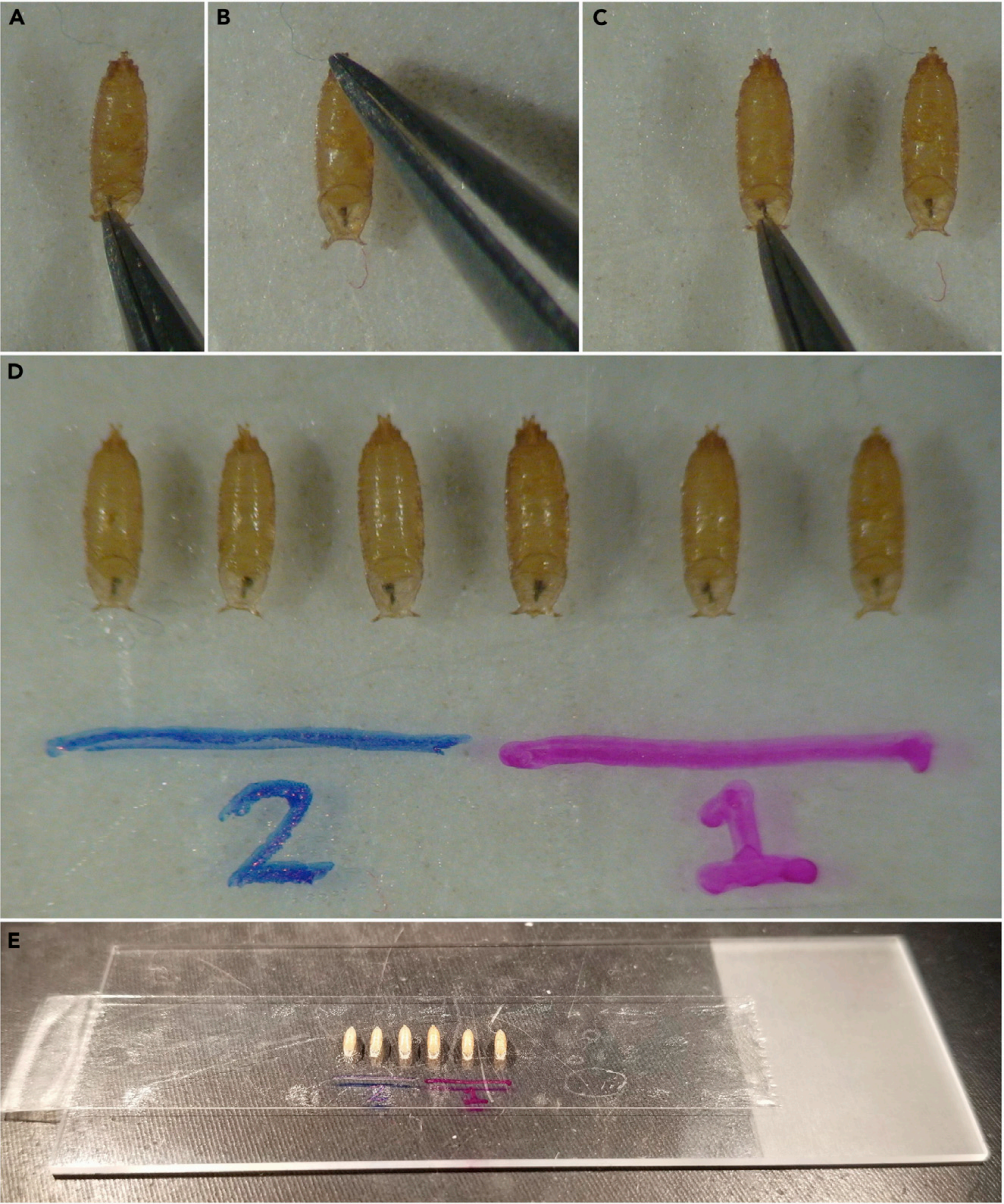
Preparation of the pupae for dissection (A) Fine-tipped forceps are used to gently grasp each pupa between the anterior spiracles, in the small gap anterior to the pupa’s head (e.g., Figure 2C, brackets), and place the pupa down on the double-sided tape. (B) Gently pressing the posterior of the pupa down on the double-sided tape aids in keeping the pupa adhered during dissection. (C) This process is repeated, evenly spacing pupae next to each other, leaving enough room for the pupal case to be dissected off and unwrapped down onto the tape between pupae. (D and E) Multiple genotypes of pupae can be prepared together, labeling the distinct genotypes if they are visibly indistinguishable.
4. With the forceps closed, press down very gently on the posterior end of the pupa to adhere it to the tape as firmly as possible (Figure 5B). If the pupae are not adhered, they will lift off the tape during dissection.
5. Place each pupa side-by-side with about two pupa-widths separating them (Figure 5C).
  a. Arrange the pupae so all the heads are approximately level with each other.
  b. If possible, arrange them with the largest pupa in the center and the smallest pupae on the ends (See troubleshooting problem 2).
6. (Optional) To dissect pupae of different genotypes on the same slide, collect all the pupae of one genotype on the tape and label with a permanent marker, then repeat steps 1–5 with pupae of another genotype (Figures 5D and 5E).

**Pause point:** If needed, this is a pause point before the dissection step, but remember the pupae are still developing.

### Dissecting the pupal case

**Timing: ∼20 min**

This section will dissect away the pupal case, revealing the head and thorax.

7. Under a dissection scope, rotate the entire slide 90° so that anterior faces right if dissecting right-handed (left if dissecting left-handed), and zoom in so the pupae are very large.
8. Use the extra-sharpened forceps to grab the operculum, gently pull it away, and discard it. It should easily pull away from the rest of the case. See Figure 6A.

**Figure 6:**
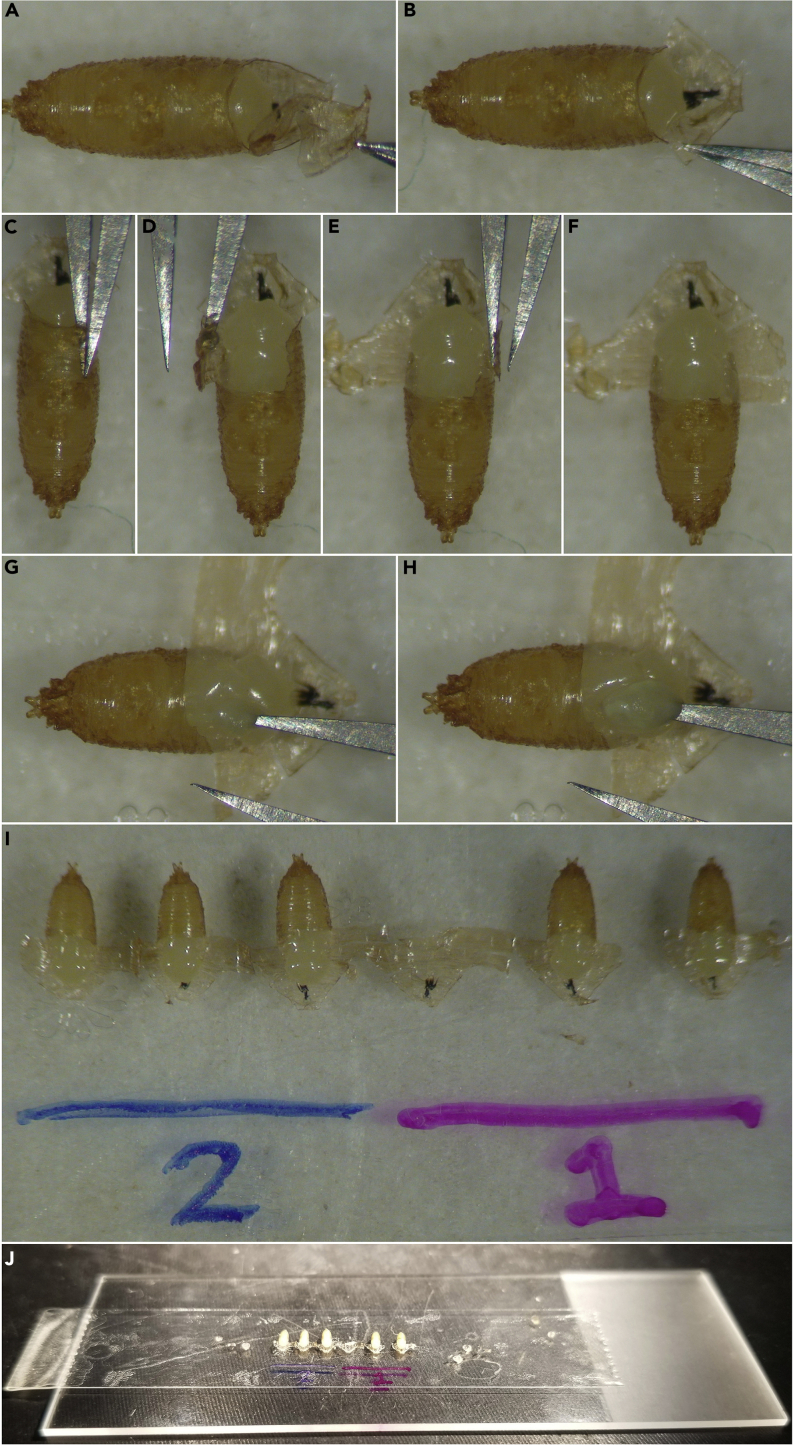
Dissecting the pupal case (A) Extra-sharp forceps grab the operculum and pull, lifting it away and exposing the pupal head. (B) The newly exposed area of the ventral case is pressed down against the tape. (C) One arm of the forceps is inserted inside the case parallel to the pupal body, lateral to the thorax and dorsal of the wing. (D) The pupal case is unwrapped over the pupa and adhered to the tape, exposing the notum. (E and F) The opposite side of the case is also pressed down onto the tape. (G and H) A puncture of the pupal body during dissection will cause the fragile epithelium to rupture, likely killing the pupa. (I and J) All pupae have had their cases dissected to expose the nota.
9. Press down the newly exposed inside area of the ventral case to the double-sided tape, Figure 6B. This will aid in the pupa remaining adhered to the tape when dissecting away the rest of the case.
10. Gently insert one tip of the forceps between case and the pupa, and slowly slide the forceps posteriorly (Figure 6C). The forceps should be inserted lateral to the midline to prevent puncturing the thorax upon entry, but medial and dorsal enough not to risk puncturing the pupal wing upon forceps insertion. Maintain the forceps at an angle parallel to the pupa and try to keep the tip of the forceps hugging the underside of the case to prevent puncturing the pupa.
11. Once the tip of the forceps is inserted approximately one-third to one-half of the way down the pupal case, gently pinch the forceps together to perforate/snip the case. Gently unwrap that portion of the case over the pupa, and adhere the unwrapped portion of the case to the double-sided tape, revealing the dorsal thorax (notum) of the pupa (Figure 6D).
12. Unwrap the remaining portion of the case on the opposite side (on the left-right pupa axis), and adhere the unwrapped portion of the case to the double-sided tape (Figures 6E and 6F).
13. Avoid puncturing the pupae during steps 10–12, as shown in Figures 6G and 6H. If a pupa is punctured, hemolymph will bleed through the epithelium and make the sample unusable. Discard and remove this pupa and continue to the next one. Do not be discouraged if this occurs multiple times when first learning this technique (see Troubleshooting problem 3).
14. Repeat this process for all pupae on the slide, see Figures 6I and 6J.

### Mounting the dissected pupae

**Timing: ∼10 min**

This section will mount the dissected pupae dorsally on a cover glass to image the notum.

15. Prepare a large cover glass (35 × 50 No. 1 used here in landscape orientation) by placing two pieces of double-sided tape on top of each other on the lower half of the cover glass (Figure 7A). The pupae will be placed so that the tape acts as a riser, lifting the abdomen and tilting the head downward onto the glass.

**Figure 7:**
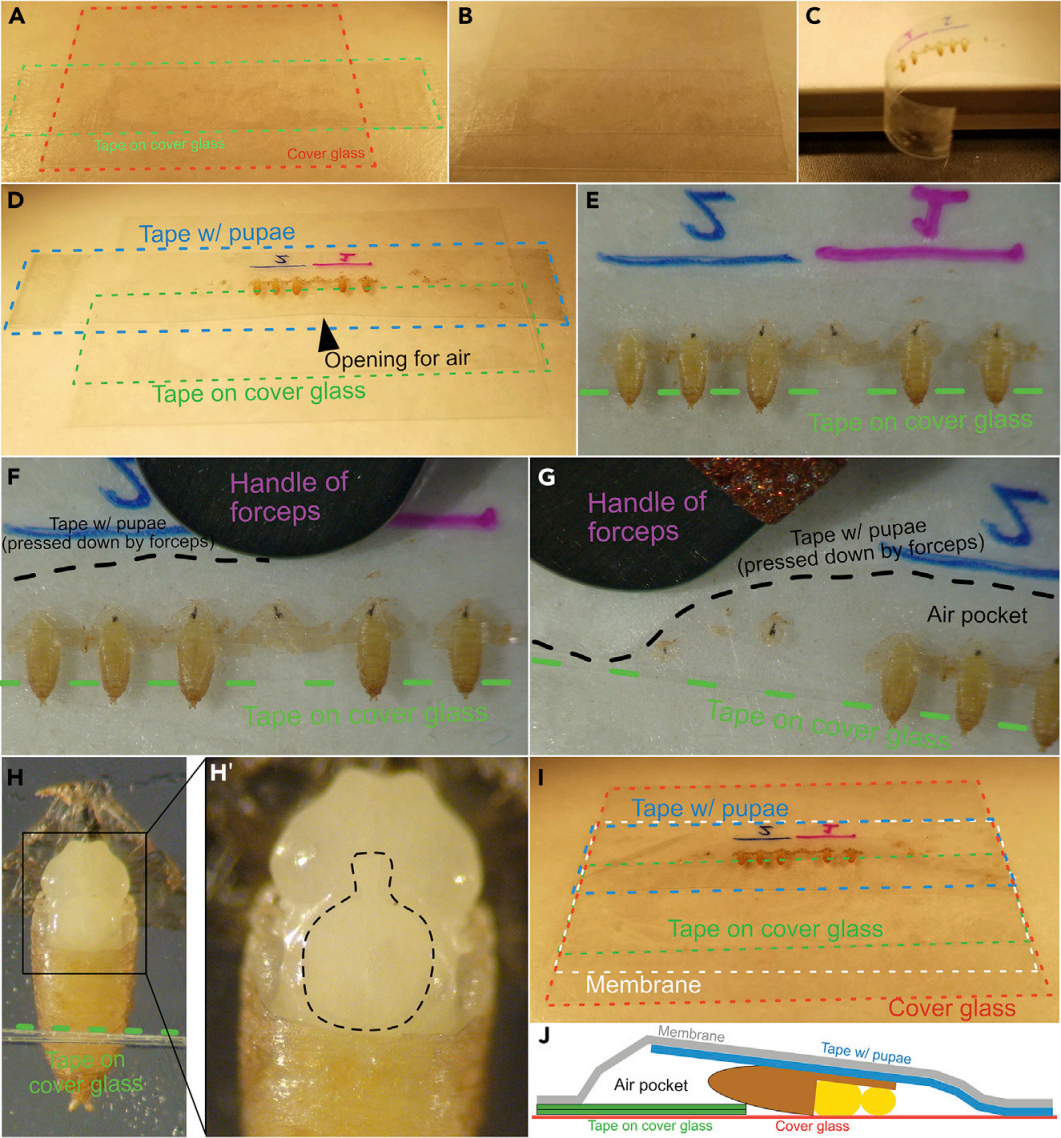
Mounting the dissected pupae (A) Two pieces of double-sided tape are placed on top of each other on the lower half of the cover glass to act as a riser/fulcrum for the pupae. (B) Overhangs are cut. (C–E) The double-sided tape with attached pupae (from Figure 6J) is removed from the microscope slide and placed on the cover glass such that the nota of the pupa touch the glass and the posterior pupal cases are laid on the riser. (F and G) The side of the tape anterior to the pupae is pressed against the cover glass using the handle of the forceps; the action of pressing down the tape anterior to the pupae combined with the fulcrum of the riser causes the notum to get pressed against the glass without harming the pupa. (H and H′) (H) A pupa with only the notum pressed against the glass (black outline, H′). (I) A semi-permeable membrane is draped over the pupae, preventing excess moisture loss while allowing gas exchange. (J) Schematic showing a lateral view of a properly mounted pupa.
16. Any overhangs can be trimmed with scissors (Figure 7B).
17. Carefully remove the double-sided tape with the pupae from the microscope slide by pulling up on the tab previously overhanging the slide (Figure 7C). Ensure the pupae are not harmed, and the tape does not curl back and stick to itself.
18. (CRITICAL) Gently lay the tape with pupae onto the cover glass with tape so that: a) the exposed nota of all the pupae are pressed against the cover glass and b) the posterior ends of the pupae are laid on the cover glass’ tape riser. The riser underneath the abdomen acts as a fulcrum, levering the head and thorax down just enough to be easily pressed directly against the glass. Do not press the tape down completely against the cover glass on the side nearest the posterior of the pupae; leave a small opening posterior to the pupae for air to enter and escape (Figures 7D and 7E). See Troubleshooting problem 4.
19. To ensure the nota are pressed against the cover glass, use the handle of the forceps to gently press the pupa-containing tape against the cover glass nearest to the anterior side of the pupae (Figure 7F), as well as on the left and right sides of the pupae (Figure 7G). As more of the tape is pressed, the head and thorax will gradually be pressed against the glass as well. The leverage gained by propping the abdomen up on the tape riser aids in tilting the head and thorax against the glass. Be careful not to press too close to the head of the thorax, for risk of popping the pupa and killing it (See Troubleshooting problem 4).
  a. Occasionally flip the slide over to check how much of the notum has been pressed down, being careful not to drop the slide and crush the pupae. The notum will have a visible “pressed against glass” effect (Figures 7H and 7H′).
20. Drape a dialysis membrane over the opening posterior to the pupae, and seal the membrane against the pieces of tape that have already been pressed down. Sealing up the sample this way continues to allow gas exchange with minimal pupal desiccation.
21. Any overhanging pupal tape or membrane can be carefully cut away (Figures 7I and 7J). The final sample can be imaged immediately or transported in an empty cover glass box.
22. When viewed under a microscope, the pupal notum should look flat and smooth. Occasionally wrinkles are observed, but these should become less prominent with practice, and generally do not interfere with imaging.
23. Pupae dissected and mounted in this manner will develop normally and about 90% survive to adulthood, even after being wounded (Antunes et al., 2013; O’Connor et al., 2021b). After eclosion, the pupae will attempt to crawl out and get stuck on the tape, demonstrating they survived to adulthood (See step 29).

### Wounding and live imaging the pupae

**Timing: ∼1 h, varies by experiment**

This section will wound the pupae by pulsed-laser ablation and simultaneously image the epithelial cells. A prerequisite is a laser ablation system attached to the microscope of choice, as described in (Kiehart et al., 2006). The following steps will demonstrate how to wound on the border of *pnr-Gal4* gene expression, as in (O’Connor et al., 2021b), using pupae of the following genotype:

*ActinP-GCaMP6m / +; pnr-Gal4, UAS-mCherry.NLS,TubP-Gal80*^*TS*^*/ +*.

24. Mount the slide on an inverted microscope for imaging.
  a. In the example shown in Figure 8A, live imaging was performed on a Zeiss LSM410 raster-scanning inverted confocal microscope with a 40×, 1.3 NA oil objective. A temperature-controlled stage may be used for long-term imaging, although we have been able to image for many hours at ambient room temperature (∼21°C).

**Figure 8:**
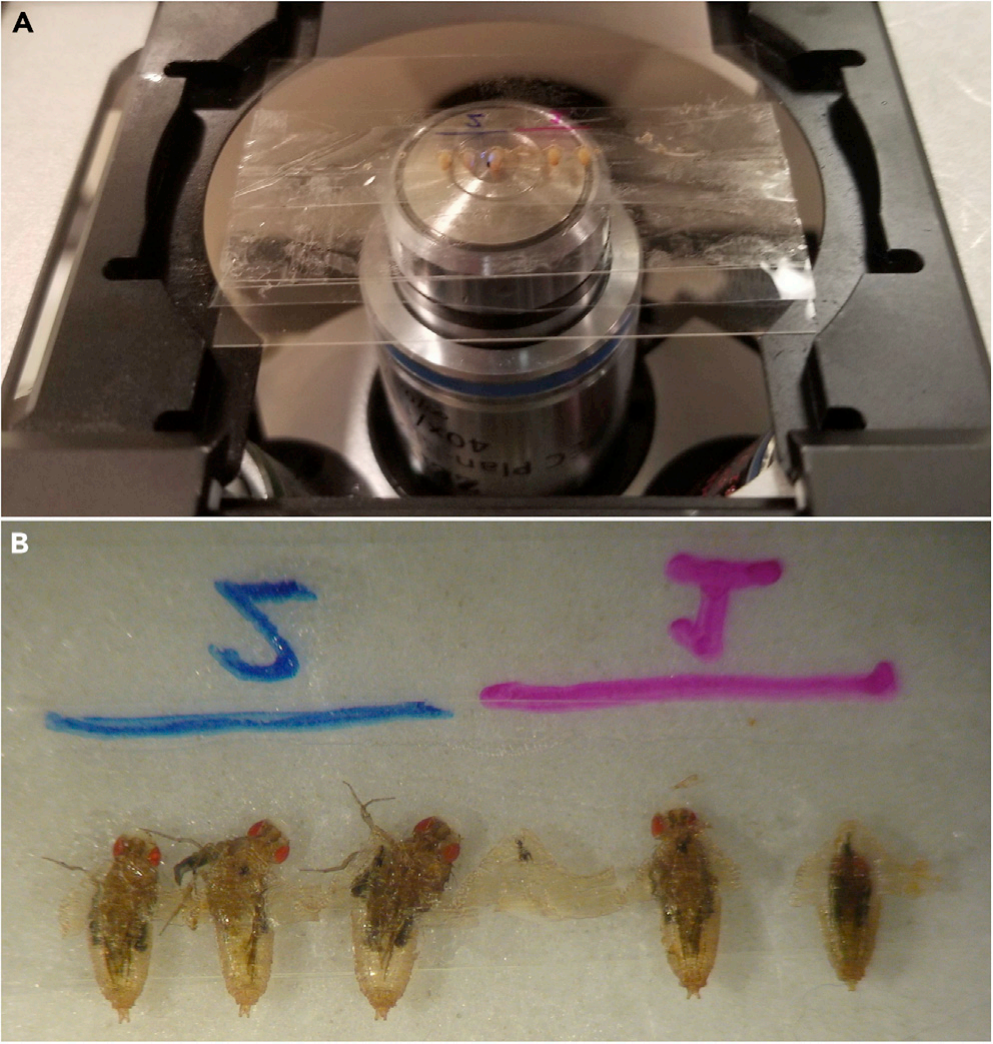
Live-imaging the pupae (A) Pupae mounted on cover glass are imaged via inverted confocal microscopy and wounded via pulsed laser ablation (see Figure 1). In the case of these pupae expressing GCaMP, epithelial calcium signaling is monitored during and immediately following wounding (see Figure 9). (B) Approximately four days later, surviving pupae eclose as adults, crawl out of their cases, and become stuck on the double-sided tape, while dead pupae never emerge from their case. Therefore, monitoring adult eclosion is a simple and effective survival assay following wounding. In this experiment, 1/2 pupae survived of genotype 1, while 3/3 pupae survived of genotype 2.
25. Move the stage to aim the laser to the desired region of the pupa. In this case, the laser ablates at the center of the frame, so the stage is aligned accordingly.
  a. Here the goal is to wound on the border of *pnr-Gal4* expression domain (indicated in Figures 4 and 9A), which is visualized by red fluorescence in the nuclei (*UAS-mCherry.NLS*)*.* Thus, we align the stage so the border of red fluorescence is in the center of the frame (Figure 9B). Set the camera settings accordingly and save this single-frame image.

**Figure 9:**
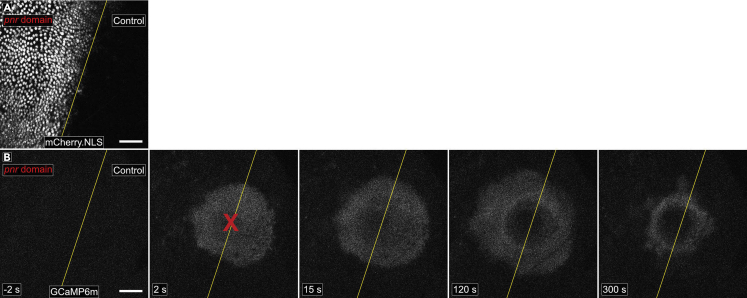
Wound-induced calcium signaling observed *in vivo* Calcium signaling data obtained after wounding using this protocol. (A) The right side is an internal control domain; the left side shows *UAS-mCherry.NLS* driven by *pnr-Gal4,* delineating the domain of *pnr* expression, which can be used to drive a variety of *UAS*-transgenes. The yellow line demarcates the border. (B) Time lapse of the ubiquitously expressed calcium sensor GCaMP6m, used to monitor calcium signaling over time following wounding. The yellow line is copied from the placement in A. Red X marks the region of ablation. Scale bar = 50 μm.
  b. We ablate pupae with a Q-switched Nd:YAG laser (5 ns pulse-width, Continuum Minilite II, Santa Clara, CA), and it is focused to ablate in the same plane as imaging.
26. To image and wound simultaneously, begin taking a time series in the relevant fluorescent channel before wounding (Figure 9C, first panel).
  a. Here the goal is to visualize intracellular calcium via the genetically encoded calcium sensor GCaMP6m, expressed ubiquitously in the pupa. In this time series, we take a frame every ∼2 s allowing for the visualization of intracellular calcium before, during, and after wounding.
27. Once baseline images are taken, ablate the sample and continue imaging.
  a. Here, the GCaMP6m calcium sensor immediately fluoresces in the cells proximal to the wound as extracellular calcium rushes into damaged cells around the wound (Shannon et al., 2017) confirming ablation has occurred.
  b. Laser pulse energies on the order of 1 μJ give wound sizes with diameter of ∼50–100 μm, although systems may vary. If desired, laser energy can be increased or decreased to change wound size (Shannon et al., 2017). Note that pulsed lasers directly kill cells at the center of the wound, and they indirectly damage cells several microns away through the laser-generated cavitation bubble (O’Connor et al., 2021a; Shannon et al., 2017).
  c. See the Troubleshooting problem 5 if the laser wound is not as large as expected, as the laser may not be in focus in the z-axis.
28. Continue monitoring the sample after wounding until the relevant biological phenomenon has been observed.
  a. Here, we observe calcium expanding from the wound for approximately 5 min after wounding (Figure 9C).
  b. See the Troubleshooting problem 6 for tips on imaging for hours.
29. Compile the time series into a movie, using a program such as FIJI/ImageJ (Schindelin et al., 2012). Annotate using the red mCherry.NLS fluorescence image to delineate the Gal4-expression domain from the internal control domain (Figures 9B and 9C, yellow lines).
30. The slide of pupae can be removed and maintained for the duration of pupal development. The pupae will complete development, eclose as adults, attempt to crawl out of their case, and get stuck on the tape. Animals that attempt eclosion are scored as viable through the end of pupal development.
  a. In Figure 8B, 1 of 2 pupae from genotype 1 (experimental) completely survived to adulthood while 3 of 3 pupae from genotype 2 (control) completely survived to adulthood.

## Expected outcomes

The outcome of this protocol is a continuous video of the notum epithelium that includes frames before and after wounding. The frame rate and the length of imaging can be adjusted to capture events that take place within seconds after wounding (see (Shannon et al., 2017) for imaging at a frame rate of 2.1 ms), events that take place over minutes after wounding (see Figure 9 with a frame rate of 2.3 s), or that take place for hours after wounding. Further, if gene knockdown or overexpression was performed in the *pnr* domain, the analysis of this data should demonstrate whether that gene has a cell-autonomous function in the process analyzed.

## Limitations

This protocol uses live imaging, so all image acquisition and analysis will depend on genetically encoded fluorescently labeled proteins. A benefit of this protocol is that imaging takes place entirely *in vivo*, without any incisions into the tissue; however, because the pupal notum is sealed by a thin transparent adult cuticle, the intact notum does not admit any tissue, cell, or nuclear stains, even those that are compatible with live imaging. To use stains and antibody reagents, the notum tissue must be dissected away from the body, fixed and stained (White et al., 2022). This same transparent adult cuticle also excludes any pharmacological agents, so the analysis of biological mechanism must rely on genetic rather than pharmacological manipulations.

Altering gene expression in the *pnr* domain is a rigorous method for analyzing gene function in a wound-related process. However, there are some important limitations. First, it is critical to establish that the response to be analyzed (here, calcium levels around wounds) is symmetric around the *pnr* border in control animals, i.e., that the response is the same within and outside the *pnr* domain when no genes are manipulated. Only once this condition is met can the symmetry of the response be used to analyze gene function by manipulating gene expression within the *pnr* domain. In the case of calcium signaling, we first established that the response was symmetric on either side of the *pnr* border (O’Connor et al., 2021b). In contrast, we have observed that the localization of blood cells to the wound is not symmetric on either side of the *pnr* border, making simple gene knockdown in the *pnr* domain insufficient to analyze gene function in this process. Another approach that might be used in cases that are not symmetric around the *pnr* border would be generating a mosaic tissue, comprised of mixed populations of clones expressing and not expressing the gene of interest (Coutiño et al., 2021).

A separate concern relevant to analyzing gene function in the *pnr* domain is whether the gene in question acts cell-autonomously, that is, will its observable action be confined to the cell that expresses it. For example, the calcium response shown in Figure 9 is mediated by a cell surface receptor, Mthl10, which in turn is activated by an extracellular ligand Gbp. Knocking down *Mthl10* in the *pnr* domain gives a clear loss of calcium signaling in that domain (O’Connor et al., 2021b); however, knockdown of *Gbp* in the *pnr* domain does not visibly alter calcium signaling, because Gbp is not produced in the notum epithelium but rather by the fat body (Tsuzuki et al., 2012). In this case, the function of Gbp can be determined by analyzing a pupa with Gbp knocked down in all cells, not just in cells in the *pnr* domain.

Related to the question of cell autonomy is the question of *pnr-Gal4* expression levels around the *pnr* border. We consider the *pnr* border to be a “sloppy border”, because the levels of *Gal4* as visualized by mCherry.NLS decline over the width of 3–4 cell diameters (Figure 9A). This gradual decline in gene expression makes it impossible to ask about phenomena in two adjacent cells that do and do not have the gene of interest. For such analysis, a clonal approach would be more appropriate (Coutiño et al., 2021).

## Troubleshooting

### Problem 1

Expression of the *UAS-Gene of interest* causes lethality before or during the pupal stage.

### Potential solution

The inclusion of *TubP-Gal80*^*TS*^ is important for cases where the gene of interest is lethal to the animal when expressed under *pnr-Gal4* expression prior to the pupal stage. *TubP-Gal80*^*TS*^ allows for gene expression to be controlled by maintaining the animals at 18°C for an extended amount of time, and minimizing the amount of time the gene is expressed at 29°C. Though it requires optimizing, some pupae that would normally die at the pupal stage can be temperature switched at the 2^nd^ or 3^rd^ instar larval stage and survive early pupal stages, and still achieve sufficient expression in the *pnr* domain for gene function to be evident by the time experimentation occurs. Additionally, knockdown or expression of genes that are embryonic lethal, but not harmful to larvae or pupae, can be performed by maintaining the P1 parents at 18°C and temperature switching the larvae to 29°C to begin gene expression after hatching.

### Problem 2

When mounting the pupae, some pupae get pressed too much and are crushed before others are sufficiently pressed enough against the glass.

### Potential solution

Spacing the pupae father apart on the slide in step 5 can help with this problem. Additionally, as mentioned in step 5b, arranging the pupae on the slide such that the largest (either by length or volume) pupae are in the center may help avoid this problem. Larger pupae will require less pressing in order for the notum to be sufficiently pressed against the glass. By extension, larger pupae will also become crushed if the tape is pressed down too much. Conversely, smaller pupae require more pressing in order for the notum to be sufficiently pressed against the glass. Therefore, a small pupa mounted in between two larger pupae will be very difficult to sufficiently press against the glass without also crushing and killing the larger pupae. Instead, by having the largest pupae in the center and the smaller pupae arranged on the sides, you can press down more on the tape to the left and right of the pupae to sufficiently press all of the pupae against the glass without over-pressing and popping the largest pupae and killing them.

### Problem 3

Difficulty in dissecting the case without killing the pupa.

### Potential solution

As mentioned in step 13, the pupae are very fragile at this stage and learning to properly remove the case can be difficult. When first learning, practice on older pupae that are less fragile and easier to dissect. Be sure the forceps are very sharp. Experiment with multiple grips that allow you to slide the forceps between the pupal body and case as consistently as possible. Finally, be sure the pupa is firmly adhered to the double-sided tape so it is not pulled off when you attempt to snip the case.

### Problem 4

Difficulty in mounting the pupae so the nota are fully pressed against the glass without killing them.

### Potential solution

As mentioned in steps 18 and 19, the two extra pieces of tape placed on the cover glass are very important to act as a fulcrum levering the head and thorax of the pupae downwards so the nota can be pressed against the glass. The pupa should be aligned with the tape on the cover glass such that only the posterior-most ∼20% of the pupa is the resting against the tape. From there, the act of pressing the tape with pupae on the side nearest the anterior of the pupae will press the head and notum down against the cover glass while the most posterior end of the pupa will remain propped in the air.

If the nota will not get pressed against the cover glass in this way, experiment with additional or fewer pieces of cover glass tape or propping the pupa further up/further down the abdomen (i.e., so that more or less of the pupa is resting against the tape).

### Problem 5

The laser did not ablate the tissue as expected.

### Potential solution

The z-focus of the laser is very important for ablation of the notum epithelia, and optimal ablation occurs when the laser is focused on the apical region of the cells. If the laser is out of focus in the z-axis, the laser may not completely destroy any cells, although the cavitation bubble may still generate damage. To aid in focusing the laser, it is helpful to have either the nuclei or the adherens junctions labeled with a fluorescent protein to aid in focusing on the apical cell surface.

### Problem 6

The sample is difficult to image over many hours.

### Potential solution

Imaging fluorescent proteins over many hours can lead to photobleaching, and this can be corrected by reducing the frame rate to collect fewer images and/or reducing the total length of imaging. A second problem that may occur over several hours is that the pupae drift out of focus in the z-axis, either due to normal development such as adult cuticle thickening slightly moving the z-plane of epithelial cells, or due to the tape holding the pupae in contact with the coverslip beginning to loosen. The sample can be manually brought into focus if long imaging is required.

## Resource availability

### Lead contact

Further information and requests for resources and reagents should be directed to and will be fulfilled by the lead contact, Andrea Page-McCaw (andrea.page-mccaw@vanderbilt.edu).

### Materials availability

This study did not generate new unique reagents.

## Data Availability

This study did not generate/analyze [datasets/code].
